# Merging microsatellite data: enhanced methodology and software to combine genotype data for linkage and association analysis

**DOI:** 10.1186/1471-2105-9-317

**Published:** 2008-07-21

**Authors:** Angela P Presson, Eric M Sobel, Paivi Pajukanta, Christopher Plaisier, Daniel E Weeks, Karolina Åberg, Jeanette C Papp

**Affiliations:** 1 Department of Biostatistics, University of California, Los Angeles, CA, 90095, USA; 2 Department of Human Genetics, University of California, Los Angeles, CA, 90095, USA; 3 Department of Biostatistics, University of Pittsburgh, Pittsburgh, PA, 15261, USA; 4 Department of Human Genetics, University of Pittsburgh, Pittsburgh, PA, 15261, USA

## Abstract

**Background:**

Correctly merged data sets that have been independently genotyped can increase statistical power in linkage and association studies. However, alleles from microsatellite data sets genotyped with different experimental protocols or platforms cannot be accurately matched using base-pair size information alone. In a previous publication we introduced a statistical model for merging microsatellite data by matching allele frequencies between data sets. These methods are implemented in our software MicroMerge version 1 (v1). While MicroMerge v1 output can be analyzed by some genetic analysis programs, many programs can not analyze alignments that do not match alleles one-to-one between data sets. A consequence of such alignments is that codominant genotypes must often be analyzed as phenotypes. In this paper we describe several extensions that are implemented in MicroMerge version 2 (v2).

**Results:**

Notably, MicroMerge v2 includes a new one-to-one alignment option that creates merged pedigree and locus files that can be handled by most genetic analysis software. Other features in MicroMerge v2 enhance the following aspects of control: 1) optimizing the algorithm for different merging scenarios, such as data sets with very different sample sizes or multiple data sets, 2) merging small data sets when a reliable set of allele frequencies are available, and 3) improving the quantity and 4) quality of merged data. We present results from simulated and real microsatellite genotype data sets, and conclude with an association analysis of three familial dyslipidemia (FD) study samples genotyped at different laboratories. Independent analysis of each FD data set did not yield consistent results, but analysis of the merged data sets identified strong association at locus D11S2002.

**Conclusion:**

The MicroMerge v2 features will enable merging for a variety of genotype data sets, which in turn will facilitate meta-analyses for powering association analysis.

## Background

Association studies for complex diseases can require thousands of samples to detect genes with small effect [1]. A minimum of 1000 to 1500 samples have been suggested for genes conferring a 1–8% increase in susceptibility to a disease, and the failure of many complex disease studies has been attributed to insufficient sample size [1-3]. As increasing the sample size for a genetic analysis improves the statistical power and therefore the ability to detect a genetic effect, complex disease studies are increasingly employing collaboration. Collaborative genetic studies often distribute genotyping among several different laboratories. If the genotyping for a family-based linkage study is distributed by assigning complete families to each laboratory (ie, all DNA samples for a particular family are genotyped at the same laboratory), logarithm of the odds (lod) scores can be computed for each data set separately and then simply added to achieve a combined lod score for the linkage study. While the analysis for distributed genotyping is often manageable for family-based linkage studies, there is no straightforward method to combine multiple association results. Consequently, an association study is best powered by combining data sets prior to analysis. If combining data sets is attempted manually without the aid of a merging algorithm, it is cumbersome at best and subject to error.

**Figure 1:**
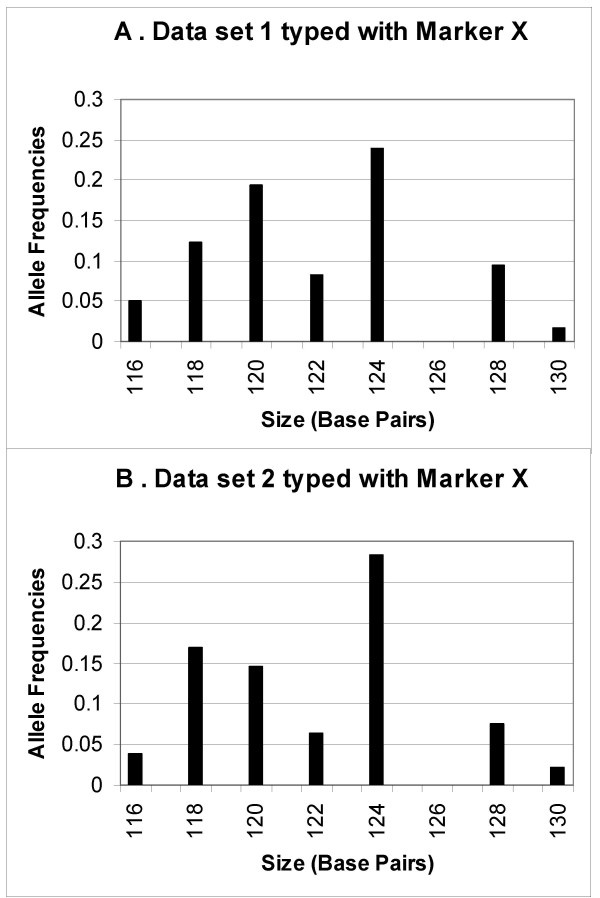
Allele frequency histograms for the same marker X typed at different laboratories on two similar sets of samples (Data set 1 and Data set 2).

In a previous publication we described statistical methodology, implemented in our software MicroMerge, for aligning microsatellite and multiallelic marker data sets [4]. MicroMerge v1 has been successfully applied by Chen et al. (2006) to linkage data [5]. MicroMerge aligns data sets marker by marker, matching each marker's allele frequencies while preserving size order. Figure 1 illustrates this alignment concept for a marker X that was genotyped on two different sets of DNA samples (data sets). An allele frequency histogram is shown for each data set, where the x-axis indicates the allele size in base-pairs, and the y-axis gives the frequency of each allele within the relevant data set. Note that seven unique alleles were observed in each data set, and the alleles had similar frequencies. We conclude that the alleles match consecutively, that is the smallest allele in data set one is the same as the smallest allele in data set two, and so on. However, in addition to allele frequency similarity, this conclusion relies on both data sets meeting the following criteria. First, each data set must consist of samples from the same ethnic group, as allele frequencies may vary among populations. Second, the sample size of each data set must be large enough to obtain accurate allele frequency estimates. In our experience, matching consecutive alleles with similar frequencies between data sets is an effective strategy for increasing genetic analysis power if the aforementioned criteria are met.

The concept of aligning microsatellite marker alleles based on frequency information rather than size (in base-pairs) was first proposed by Dorr et al. in 1997 [6]. In 2002, Weeks et al. illustrated the danger of aligning data sets using base-pair size information alone by sending the same set of DNA samples to two different genotyping facilities [7]. When these two data sets were merged marker by marker, according to allele sizes, the resulting merged data set had a genotype error rate of 16.8%. This is because allele sizes are only estimates that can differ between laboratories, instruments or experimental protocols. The consistency of estimating allele size within a particular facility, platform and protocol enables precise grouping of similarly-sized DNA fragments into a 'bin', which represents an allele. There are multiple methods for defining these bins, so that different grouping strategies can alter allele names for the same raw genotype data [8]. For a more thorough discussion of the difficulties of merging microsatellite data and prior research in this area, we refer the reader to our previous MicroMerge publication [4].

**Figure 2:**
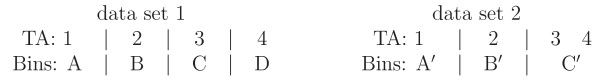
**Example of a MicroMerge alignment for two data sets.** Data set 1 has four bins, data set 2 has three bins and the data sets are aligned to four theoretical alleles (TA).

For reasons mentioned above, allele calls may only approximate the true alleles present in a set of DNA samples. MicroMerge uses a Bayesian model to align the observed bins to a proposed set of true theoretical alleles, where the number of theoretical alleles is estimated from the model. For clarity we will use the term 'bin' to distinguish an observed data set allele from an actual theoretical allele. MicroMerge uses MCMC methods to sample from the posterior distribution of possible alignments given the genotype data. Each MicroMerge alignment is characterized by three parameters (1) the number of theoretical alleles, (2) the theoretical allele frequencies, and (3) the partition. Figure 2 gives an example of a partition aligning bins from two data sets to four theoretical alleles. The vertical bars in this figure indicate correspondence between the data set bins and theoretical alleles. For example, bin *A *in data set 1 aligns with theoretical allele 1, and bin *C*' in data set 2 aligns with both theoretical alleles 3 and 4. On each iteration of the MCMC chain a change is proposed to one of the three alignment parameters, and the proposal is accepted or rejected by comparing the likelihoods of the proposed and current states.

The focus of this paper, like our previous MicroMerge paper, is on merging multiallelic marker data (where microsatellite markers are the most common type of multiallelic markers). Merging multiallelic marker data sets is more challenging than merging SNP data sets because multiallelic markers may have ten or more alleles of varying fragment lengths. In comparison, a SNP marker usually has only two alleles whose identities are often known. In the Conclusions section we describe how MicroMerge might be applied to SNP data sets. But, in spite of the increasing popularity of SNP data, multiallelic markers continue to be widely used for genetic analysis. In a survey of annual publication records in the NCBI PubMed database  since the year 2000 there have consistently been more than 3600 articles per year containing keywords pertaining to multiallelic markers (microsatellite marker, di/tri/tetranucleotide repeat, short tandem repeat, variable number tandem repeat and their plural and acronym forms). In comparison, the number of articles in a single year containing keywords pertaining to SNP markers (SNP, single nucleotide polymorphism and their plural forms) did not exceed 3600 articles until 2005. In the last three years (2005–2007) there were 5657, 5825 and 5745 annual multiallelic marker articles, and 4287, 5180 and 6508 SNP articles. While there is clearly an increasing trend in the number of SNP articles, the multiallelic marker articles have been holding constant over the past three years and have only been outnumbered by SNP articles in the past year. As a result, the importance of this marker type should not be discounted by the increasing interest in SNP data.

While MicroMerge v1 was able to successfully merge and create output files which could be analyzed by some programs such as Mendel, we realized a need for more flexible merging options [9]. MicroMerge v2 includes seven new features that significantly extend the capabilities of v1. In the next sections we describe these features and present results from both simulated and real data sets. Finally, we show how MicroMerge can increase the statistical power of a genetic analysis by comparing association results from three separately analyzed familial dyslipidemia data sets to the association result obtained from analyzing the combined data sets.

## Methods

Before describing the new MicroMerge features and their underlying methodologies, we define some terminology and introduce the data sets used to test MicroMerge. "Overlap" and "distance" are two measures of alignment accuracy that were described in our previous publication [4]. Overlap is a measure that quantifies the certainty of each bin pair within an alignment. A bin *i *defined by lab 1 and a bin *j *defined by lab 2 overlap if they both align with one or more of the same theoretical alleles. The overlap probability for this pair *o*_*ij *_can be approximated by the fraction of the sampled alignments where overlap occurs. The "average overlap" for an alignment *A *is just the average of the overlap probabilities for each bin pair within the alignment.

"Distance" quantifies the accuracy of a MicroMerge alignment in relation to a manually obtained alignment. Each manual merge was obtained by aligning data set bins marker by marker using frequencies, relative base-pair size differences, eight samples common to both data sets, and aligning the data by eye. Consider a binary indicator overlap matrix *L *for an alignment *A*, where the dimensions are defined by the number of bins in each data set. Unit entries *l*_*ij *_in *L *indicate whether bins *i *and *j *overlap in alignment *A*.

The distance between a MicroMerge alignment *A *and manual alignment *M*, is given by

$$||A-M||\;=\sum_{i}\sum_{j}\left|l_{ij}(A)-l_{ij}(M)\right|.$$

Note that we only report alignment accuracy (distance) results when a reference alignment is available. The MicroMerge v1 algorithm ascribed differences in observed bins for a particular marker between data sets typed at different laboratories to allele-calling differences. This assumption often produces 'lumped' alignments where a bin from one data set may align with more than one bin in another data set. While it is more flexible than forcing a bin from one data set to align with only one bin in another data set, or creating a 'one-to-one' alignment, the resulting merged data files pose a problem to most genetic analysis programs. This is because if a bin in data set 1 aligns with two bins in data set 2, their relationship is coded as a phenotype in the merged locus and pedigree files. In order to generalize the output files so that they can be understood by the majority of genetic analysis programs, we include an option to create merged files with one-to-one alignments. Interpretations of the two alignment types are described in the following two sections.

### Lumped alignment

A lumped alignment assumes that for a particular marker all of the same unique bins exist in both data sets, but that genotyping differences or genotyping errors may have caused the reported number of unique bins to differ between the data sets. For example, data set 1 in Figure 2 observes four bins, but data set 2 observes only three bins because theoretical alleles 3 and 4 are lumped into bin *C*'. In the pedigree file for the above example, the *C*'/*C*' genotype in data set 2 would be coded as a phenotype 2: *C*' + *C*', where "2" corresponds to data set 2, and the bin names are included for clarity. The 2: *C*' + *C*' phenotype would be consistent with any of the following genotypes: 3/4, 3/3, and 4/4, and this correspondence would be included in the locus file. The data set resulting from this alignment includes genotypes expressed as non-codominant phenotypes, and must be analyzed by capable software such as Mendel [9].

### One-to-one alignment

**Figure 3:**
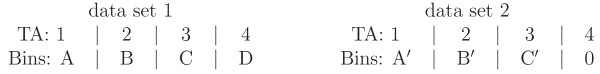
**Example of a one-to-one MicroMerge v2 alignment for the data sets in Figure 2.** In this case *C*' in data set 2 corresponds only with bin *C *in data set 1. TA is an abbreviation for "theoretical alleles".

The one-to-one alignment format explains discrepant numbers of bins between data sets for a particular marker by assuming that an allele observed in one data set is missing from the other data set. Figure 3 shows a one-to-one alignment similar to the lumped alignment from Figure 2, except that *C*' in data set 2 was paired with only theoretical allele 3, and thus with only bin *C *in data set 1. The zero indicates that bin *D *in data set 1 was not present in the data set 2 DNA samples, and there were no '4' alleles observed in that sample. The *C *= *C *genotype in data set 2 would correspond to a single genotype 3/3 in the merged locus and pedigree files, hence the merged one-to-one format data can be analyzed by any program that handles genotype data such as GENEHUNTER [10], MERLIN [11] and LINKAGE [12]. We detail how the one-to-one alignment is translated from a lumped alignment after describing the test data sets.

### Test data sets

We tested the accuracy of our new MicroMerge v2 features on both simulated data and the real genotype data that was described in our previous publication, which we refer to as the 'real data project' [4]. In the following Methods and Results and Discussion subsections the test data was simulated unless otherwise noted.

The real data project consisted of a pair of data sets with approximately 87 and 333 samples, typed with 50 microsatellite markers. The correct alignment between these data sets was known for 48 of the markers based on a manual merge, where each manual alignment was obtained by eye without the aid of a computer or merging algorithm (as previously described). In order to assess the accuracy of both lumped and one-to-one alignment formats, a corresponding one-to-one reference alignment was manually translated from each lumped manual merge alignment. "Bin spacing" information refers to the base-pair differences between bins of consecutive size. While the actual base-pair size is unreliable, generally genotyping is consistent within a laboratory. For example, a dinucleotide marker typically has bins spaced in increments (or multiples) of approximately two base-pairs.

The lumped and one-to-one alignments require different manually obtained alignments. After manually obtaining a lumped alignment by eye, we then manually selected the corresponding one-to-one alignment with the best allele frequency match between the data sets. In a few cases it was difficult to determine the best manual alignments. For example, if the number of unique alleles differed by ≥ 2 alleles between data sets.

We use a familial dyslipidemia study sample containing dinucleotide repeat microsatellite genotypes from chromosome 11 markers to illustrate MicroMerge v2's ability to improve association analysis. The familial dyslipidemia study sample consists of Dutch (*Dutch*) and two Finnish (*Finn*1 and *Finn*2) data sets genotyped with the Weber screening set version 6 for the purpose of studying familial dyslipidemia (FD) [13,14]. The *Dutch *data set consisted of 275 samples genotyped on Applied Biosystems' ABI PRISM^® ^377 platform. The *Finn*1 data set comprised 228 samples genotyped at the UCLA Genotyping and Sequencing Core on LI-COR 4200 DNA Analyzers. *Finn*2 consisted of 248 samples genotyped at the National Public Health Institute of Finland on Applied Biosystems' ABI PRISM^® ^377 platform. We considered a set of ten markers D11S1984, D11S2362, D11S1999, D11S1981, D11S1392, D11S2002, D11S2000, D11S1998, D11S4464 and D11S912 that were genotyped on all three data sets. The range for the number of unique bins per marker in the *Dutch *data set was 6–18 (with an average of 8.9 bins per marker), 6–13 (with an average of 7.9 bins per marker) in the *Finn*1 data set and 6–17 (with an average of 8.8 bins per marker) in the *Finn*2 data set. On average the number of unique bins per marker differed by one between data sets. Marker D11S200 had the greatest difference in number of unique bins observed among the data sets with 18 unique bins in the *Dutch *data set, 13 unique bins in *Finn*1 and 17 in *Finn*2.

Now that we have covered some terminology and described the test data sets, we move to details of the MicroMerge v2 features. In the remainder of this section and in the following Results and Discussion section, each new MicroMerge v2 feature is numbered and titled for clarity. To assist the reader, we have also summarized these features and their usage in Table 1.

**Table 1:** Overview of the new features incorporated into MicroMerge v2 and guidelines for when to use them

**New Feature**	**Purpose**	**Guidelines and Usage**
**Feature 1: One-to-one alignment format**	Creates flexible output files and provides a more suitable alignment format for most data sets. Both one-to-one and lumped alignment formats are available in v2.	The lumped format may be preferred for data sets that will be analyzed with Mendel and have 1) few rare bins (<5–10% of bins with <6 instances), 2) discrepant numbers of unique bins between data sets (most markers differ by 2–3 bins), 3) genotyping that was done on platforms with different resolving power, or 4) other situations where bin frequencies don't match well. Otherwise, use the default one-to-one format.
**Feature 2: Controlling one-to-one alignment translation**	Allows > 1 one-to-one translation from each lumped alignment.	This feature was not useful for our test data sets but can potentially increase alignment posterior probability for markers that have many one-to-one translations with competitive likelihoods. The default value is 1 one-to-one alignment translation per lumped alignment.
**Feature 3: Re-merging markers with low posterior probabilities**	Improves alignment of markers that have low posterior probabilities and rare bins by zeroing these bins and re-merging the data. Results in a second set of merged data files.	There are three parameters controlling marker selection for re-merging: 1) alignment posterior probability (< 0.425) and bin pair(s) that 2) have low overlap (< 0.85) and 3) low theoretical allele frequencies (< 0.015). The user can control the frequency of re-merging by adjusting these three parameters (from the above default values) in the control file.
**Feature 4: Adjusting the prior on the theoretical allele number**	Controls emphasis on alignments that have fewer theoretical alleles.	Allows *τ *to range from 0.05–0.3 (where 0.2 is the default), corresponding to decreasing emphasis on alignments with fewer alleles. Smaller *τ *values are useful when one or more data set(s) are several times larger than the other(s).
**Feature 5: Using population allele frequencies to align data**	Improves alignment of data sets with unreliable allele frequencies.	Enables alignment of small data sets and data sets from different ethnic groups. If reliable population allele frequencies are available then this feature should be used to improve alignments.
**Feature 6: Aligning multiple data sets**	Allows simultaneous alignment of >2 data sets.	All data sets should be merged simultaneously.
**Feature 7: Likelihood ratio test (LRT) for assessing alignment quality**	Provides another measure of alignment quality that is more general than the posterior probability.	Applicable to lumped alignments only, > 90% of lumped alignments should reject the LRT null hypothesis (LRT = 1).

#### Feature 1: One-to-one alignment format

**Figure 4:**
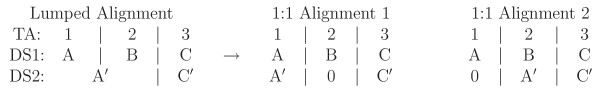
Example of a lumped MicroMerge alignment and its corresponding one-to-one alignments, where TA = theoretical alleles, DS1 = data set 1 bins, and DS2 = data set 2 bins.

To retrieve a one-to-one MicroMerge v2 alignment, the original MicroMerge algorithm is preserved, but the lumped alignments are translated to one-to-one alignments. In both Micromerge versions lumped alignments are saved every 1,000 iterations after a burn-in period, and a default value of 1,000 alignments are saved to determine the alignment(s) between the data sets. When the user selects the one-to-one alignment option, each lumped alignment is translated to all possible corresponding one-to-one alignments. The default setting chooses the one-to-one alignment with the highest likelihood to represent the lumped alignment. The likelihood is computed from the product of the data set genotype frequencies, where the genotype frequencies are computed assuming Hardy-Weinberg equilibrium values and the bin frequencies are obtained from the theoretical allele frequencies. The frequency of each zeroed theoretical allele is divided among the remaining alleles. Figure 4 shows an example of the two one-to-one alignments corresponding to a lumped alignment with three theoretical alleles.

#### Feature 2: Controlling one-to-one alignment translation

The default setting for the one-to-one alignment option selects the most probable one-to-one alignment translation for each lumped alignment. We allow some control over this translation process to offer flexibility when a lumped alignment has many one-to-one translations with competing likelihoods. In the case where *X *one-to-one alignment translations are saved for each lumped alignment, each one-to-one alignment *t *is weighted by its likelihood with weight $w_{t}=\frac{L_{t}}{\sum_{v=1}^{X}L_{v}}$. *L*_*t *_is the likelihood of alignment *t *and for each set of *X *alignments $\sum_{t=1}^{X}w_{t}=1$ so that for each lumped alignment the count of saved one-to-one alignments is incremented by one.

#### Feature 3: Re-merging markers with low posterior probabilities

A limitation of MicroMerge v1 was that about 20% of markers had low posterior probability alignments, and we recommended against analyzing the merged results for these markers. These low posterior probabilities were often due to frequency differences among rare bins. To see how this problem could arise, consider a rare bin occurring once in one data set, and twice in another data set of the same size. While this could easily happen by chance, bin frequencies differing by a multiple of two or more are considered to be substantially different. In this case the presence of one or two rare alleles can result in a low posterior probability, rejection of the alignment, and the loss of data for an entire marker.

In MicroMerge v2 we developed a method for zeroing rare bins and re-merging the data sets for qualifying markers. When a marker's top alignment posterior probability falls below a threshold, it is considered for re-merging. Then if a pair of aligned bins (or set of aligned bins in the case where more than two data sets are merged) has an overlap probability below a threshold value and its corresponding theoretical allele frequency qualifies as rare, the theoretical allele is zeroed. Zeroing a theoretical allele means that all corresponding data set bins with only one instance in the alignment (ie, bins having a one-to-one correspondence with the zeroed theoretical allele) are zeroed in their pedigree files. The data for this marker are then re-merged.

Table 2 shows an example of executing this method for marker D4S291 from the real data project. MicroMerge v2 had aligned D4S291 correctly based on a manually obtained alignment but had rejected it for merging due to a low alignment posterior probability. Imposing a minimum overlap probability of 0.85 and minimum theoretical allele frequency of 0.015, the theoretical alleles 5, 6 and 8 are zeroed. These theoretical alleles correspond to E', F' and H' in data set 2 and bins E and F in data set 1. The data set 2 bins all align uniquely to the zeroed theoretical alleles. As a result, all genotypes containing either E', F' or H' are removed from the data set 2 pedigree file. Since there are multiple instances of both E and F in the data set 1 alignment, there is no change to the data set 1 pedigree file. Marker D4S291 is then re-merged to achieve a more confident alignment. We demonstrate this option on the 48 real data markers and simulated data sets for both lumped and one-to-one alignment formats.

**Table 2:** Example of a top alignment for marker D4S291 with low posterior probability 0.235 due to four consecutive low frequency alleles (theoretical alleles 5–8).

Data 1	Data 2	TA	TF	Overlap Probability
A	A'	1	0.5442	1.000
B	B'	2	0.0281	0.997
C	C'	3	0.0453	1.000
D	D'	4	0.2859	1.000
E	E'	5	0.0104	0.552
E	F'	6	0.0075	0.806
E	G'	7	0.0198	0.798
F	H'	8	0.0114	0.698
F	I'	9	0.0473	1.000

TA is an abbreviation for "theoretical alleles" and TF is an abbreviation for "theoretical frequencies".

#### Feature 4: Adjusting the prior on the theoretical allele number

Another enhancement of MicroMerge v2 is an option allowing control over the number of theoretical alleles. This is useful for situations where data set bin frequencies do not match well, but it is expected that the same bins have been observed within each data set. For example, if one data set is several times larger than the other, it seems likely that the larger data set would observe all bins present in the smaller data set. In this scenario, it may be useful to put stronger emphasis on alignments with fewer theoretical alleles. The number of theoretical alleles *n *is allowed to vary between the maximum number of unique bins observed in a single data set (*n*_min _= max_*i*_|**B**_*i*_|, where |**B**_*i*_| is the number of unique bins observed in data set *i*) to the sum of all unique data set bins ($n_{\max}=\sum_{i}^{m}\left|B_{i}\right|$). The geometric prior distribution of *n *with success probability *τ*

$$\frac{\tau^{n}}{\sum_{m=n_{\min}}^{n_{\max}}\tau^{m}}=\frac{\tau^{n}(1-\tau )}{\tau^{n_{\min}}-\tau^{n_{\max}+1}}$$

encourages smaller values. In MicroMerge v2 the user can adjust the default value for *τ *within a reasonable range. While all allowable values for *τ *emphasize alignments with a small number of theoretical alleles, this option allows control over the strength of this emphasis.

We test this option on the real data, and present results for several selected markers. Results are reported for markers with a) top alignments containing potentially excess theoretical alleles (*n *= *n*_*min *_+ 1), b) similar posterior probabilities among the top alignments and c) incorrect alignments. The idea behind this test was to show that while the minimum value might increase the posterior probability of set b) and increase the accuracy of set c), it could weaken results for set a).

#### Feature 5: Using population allele frequencies to align data

If a data set is too small to obtain accurate estimates of bin frequencies, the user can now merge the data using specified allele frequencies rather than allele frequencies estimated from the data set. When reliable allele frequencies of ethnically matched populations are available, these population frequencies can be substituted for the calculated bin frequencies of any data set(s) to be merged. We implement this feature by listing population frequencies in the locus file(s) and alerting MicroMerge v2 with an option in the control file to use these frequencies. These population frequencies are then used to generate a pedigree file that is used in place of the original pedigree file to align the data sets. Once the optimal alignment is known, the original pedigree files are merged. This feature would also be useful for users who wish to simulate pedigree files based on a given sample size and locus file.

MicroMerge v2 allows some control over the construction of the generated pedigree file. The default sample size is equal to the average size of the other data sets (with a minimum value of 200 samples), but the user can specify an alternative sample size using this Feature 5 option. In addition to choosing the sample size(s) the user also has a choice between two different data generating schemes. The nearly exact number of genotypes can be computed from the predicted genotype frequencies under Hardy-Weinberg equilibrium (HWE), or each genotype can be sampled according to the HWE genotype frequencies. The data generating process is controlled by an option in the control file, where the default method is to sample from the genotype frequencies.

This option was tested for the one-to-one alignment format on six real data markers. Half of these markers had incorrect alignments when the bin frequencies were estimated from the pedigree files. The larger data set bin frequencies were used as population frequencies for the smaller data set to demonstrate the utility of this option.

#### Feature 6: Aligning multiple data sets

In the case where there are more than two data sets, MicroMerge v2 is able to merge them simultaneously (whereas MicroMerge v1 had to align them in a pair-wise fashion). Simulated data sets were used to test the success of aligning multiple data sets simultaneously. Table 3 shows the population bin frequencies that were used to create the three simulated data sets *D*_*a*_, *D*_*b*_, and *D*_*c*_. *D*_*a *_consisted of 100 samples and observed all four population bins. *D*_*b *_and *D*_*c *_both had a sample size of 50 and were missing one bin each. We tested the multiple data set alignment feature by first merging *D*_*b *_with *D*_*c*_, merging the resulting data set with *D*_*a*_, and then contrasting this alignment result with the simultaneous alignment of data sets *D*_*a*_, *D*_*b*_, and *D*_*c*_. This test was conducted on both the lumped and one-to-one alignment options.

**Table 3:** Three simulated data sets each consisting of *N*_*i *_genotype samples from the given distribution of population allele frequencies.

Population Alleles	Allele Frequencies	Data *A *(*N*_1 _= 100)	Data *B *(*N*_2 _= 50)	Data *C *(*N*_3 _= 50)
1	0.005	*A*_1_	*B*_1_	0
2	0.010	*A*_2_	0	*C*_2_
3	0.185	*A*_3_	*B*_3_	*C*_3_
4	0.800	*A*_4_	*B*_4_	*C*_4_

These data sets were used to test the success of aligning multiple data sets simultaneously. Data set *B *was aligned with data set *C*, and then the combined data set was aligned with data set *A*. This alignment was then compared to the simultaneous alignment of data sets *A*, *B*, and *C*.

#### Feature 7: Likelihood ratio test for assessing alignment quality

Three alignment quality assessment measures were presented in the first MicroMerge release: posterior probability, average overlap, and the difference between the posterior probabilities of the top two alignments. We include an additional score in MicroMerge v2, based on a maximum likelihood criterion. We implement a likelihood ratio test by comparing (1) the likelihood computed using sample counts as the bin frequency estimates to (2) the merged data likelihood, where the data set bin frequencies are derived from the theoretical allele frequencies. The likelihood ratio statistic [15] is 2*ln*(*Q*_0_/*Q*_1_), where *Q*_0 _is the merged data likelihood and *Q*_1 _is the original data likelihood. This statistic follows the chi-square distribution *χ*_*v*_, with degrees of freedom *v *equal to the difference in the number of independent parameters between the two models $\sum_{j}^{d}\left|B_{j}|-(n-1\right)$ (where *d *is the number of data sets and |*B*_*j*_| is one less than the number of unique bins in data set *j*, and *n *is the number of theoretical alleles).

#### Association analysis of familial dyslipidemia study sample

We illustrate MicroMerge's utility for association analysis using the familial dyslipidemia (FD) study samples. For both lumped and one-to-one formats, we created merged data sets for all three FD data sets and all pair-wise combinations of the FD data sets, resulting in a total of eight different merged data sets. We then ran Mendel's Option 8 gamete competition analysis [9] on each data set independently and on the eight combinations of merged data sets. The gamete competition option was selected for its ability to handle the phenotype data generated by bin lumping.

## Results and discussion

For the results presented here, **Feature 3: re-merging markers with low posterior probabilities **was run in combination with each of the other MicroMerge v2 features. This option creates the standard set of output files as well as an additional set of files for any markers that were re-merged. Because Feature 3 expands MicroMerge v2's merged data output, we recommend using a more conservative posterior probability threshold of 0.425 with this option (for lumped alignments) rather than the previously published 0.3 threshold for MicroMerge v1. Both of these thresholds were empirically determined based on the accuracy of the real data project alignments in comparison to the manually-obtained alignments. The results described below are based on this more conservative threshold for defining acceptable alignments.

### Feature 1: One-to-one alignment format

**Figure 5:**
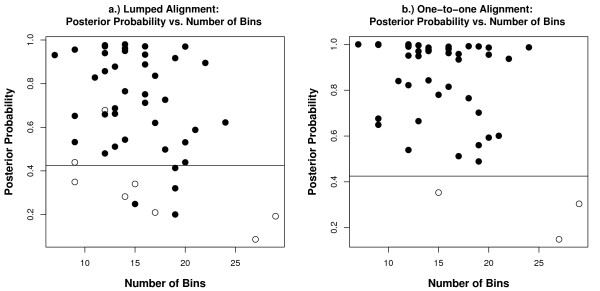
**Accuracy of MicroMerge v2 alignments for the 48 real data project markers, as compared to a manually obtained reference alignment for the lumped alignment format (a) and the one-to-one format (b).** The manual alignment was obtained by eye (without the aid of a merging program) by using bin frequencies, relative base-pair size differences, and eight samples common to both data sets. Each plot shows the posterior probability of each marker plotted against its number of unique bins (summed from both data sets), where a black circle indicates agreement between the MicroMerge v2 alignment and its corresponding reference alignment and a white circle indicates disagreement. The horizontal line at 0.425 represents the same acceptance threshold for comparison between the methods. For the real data project the one-toone alignment format (b) is more accurate than the lumped alignment (a) because all correct MicroMerge v2 alignments had posterior probabilities above the threshold and only three markers were misaligned. In comparison the lumped format had four correctly aligned markers with posterior probabilities that fell below the threshold and eight markers total were misaligned.

In order to test the success of the one-to-one alignment format, we compared the accuracy results for both the one-to-one and lumped formats for the 48 real data project markers. Plots of the top alignment posterior probabilities versus the number of unique data set bins are shown in Figure 5 for both formats, where markers with top alignments in disagreement with the manual alignment are indicated with white circles. The lumped alignment format resulted in eight markers with discrepancies between the MicroMerge and manual alignments (Figure 5a), whereas the one-to-one format had only three (Figure 5b). The 0.425 posterior probability threshold discarded ten markers in the lumped format of which only six had alignments in disagreement with the manual alignment. This threshold also failed to flag two markers with small distances of one and two from the manual alignment. The 0.425 threshold discarded all three one-to-one format alignments that conflicted with the manual alignment. While the one-to-one format performed better for this data set in terms of the number of accurately aligned markers, the lumped alignment distance was lower on average (3.5/marker versus 6.7/marker in the one-to-one format).

### Feature 2: Controlling one-to-one alignment translation

As mentioned in the Method's section, the one-to-one alignment is translated from the lumped alignment, and each lumped alignment may have many possible one-to-one alignment translations. The default setting is to choose the highest likelihood translation, but we allow the user to save more than one translation per lumped alignment.

We tested this option by saving the top three (*X *= 3) one-to-one alignment translations from each lumped alignment for the real data project markers. The alignment for D4S3042 was marginally improved from a distance of 10 to 8, but this alignment also had a low posterior probability, so this improvement could be due to chance. While saving only one one-to-one alignment per lumped alignment performed best on our test data sets, the user has the option of testing this feature.

### Feature 3: Re-merging markers with low posterior probabilities

Table 4 shows the results for re-merging the lumped alignments from the real data project. *A*_*o *_refers to the top 'original' alignment result for the first merge and *A*_*d *_is the top alignment result for the re-merged data. Since data set bins were zeroed before re-merging, accuracy was gauged using a manual alignment with the same bins zeroed *M*_*d*_.

**Table 4:** MicroMerge v2 lumped alignment results for nine poorly aligned markers before and after remerging.

	# Bins		Original		Re-merged		
Marker	Lab1	Lab2	Pr(*A*_*o*_)	||*A*_*o *_- *M*_*o*_||	Pr(*A*_*d*_)	||*A*_*d *_- *M*_*d*_||	# Zeroed
D4S2961	6	9	0.248	0	0.958	0	3
D4S250	9	10	0.413	0	0.957	0	1
D4S2460	6	8	0.282	4	0.606	2	1
D4S1637	7	8	0.34	5	0.539	3	1
D4S3043	8	9	0.209	2	0.529	0	1
AFMA082Y	8	11	0.32	0	0.509	0	1
D4S2380	11	16	0.086	6	0.509	2	3
D4S3042*	**13**	**16**	**0.192**	**6**	**0.386**	**3**	**8**
D4S1560*	**8**	**11**	**0.2**	**0**	**0.339**	**0**	**0**

Average		8.92	0.25	2.83	0.61	1.17	1.67
Total		107		17		7	10

*A*_*o *_is the original MicroMerge alignment, *M*_*o *_is its corresponding manually obtained alignment, *A*_*d *_is the re-merged Micromerge v2 alignment, and *M*_*d *_is its corresponding manually obtained alignment.

*Posterior probability remained low after dropping problematic alleles and thus these markers' values were excluded from the 'Average' and 'Total' summaries.

Ten markers were rejected due to low alignment posterior probabilities (< 0.425). Nine of these markers also had at least one rare theoretical allele (with a frequency < 0.015). MicroMerge v2 zeroed the data set bins corresponding to these low frequency theoretical allele(s) if the overlap probability was also low (< 0.85). After re-merging, seven markers had top alignment posterior probabilities exceeding the minimum threshold of 0.425 (see unshaded markers in Table 4). In all cases, the MicroMerge v2 alignment agreement with the manual alignment either improved (five markers) or remained the same (four markers). Only two markers continued to have alignment posterior probabilities below the default threshold (shaded grey in Table 4). The re-merge option improved the yield of merged bins from 75% to 88%, with only a 1% reduction in accuracy to 98.5% (where accuracy was defined as the percentage of correctly aligned bins in comparison to a manually obtained alignment). For comparison, merging all of the data had an accuracy of 96.2%.

While this feature worked well for lumped alignments, it was less successful for the one-to-one format. According to the same lumped format criteria, three low posterior probability markers in the one-to-one alignment format were eligible for re-merging. D4S1637 and D4S3042 were re-merged correctly with posterior probabilities greater than 0.6 (where about 20% of bins were zeroed in both cases), but D4S2380 was less accurate after re-merging. The distance for this marker increased from 10 to 13, while the posterior probability also increased from 0.149 to 0.471. If the re-merging option is selected for the one-to-one alignment format, it may be useful to raise the accuracy threshold to about 0.5. In the real genotype data project the one-to-one format aligned 90.4% of bins with 100% accuracy, outperforming the lumped alignment format which aligned 88% of bins with 98.5% accuracy. When all data is merged with the one-to-one method, the overall accuracy is still high at 97.3%.

### Feature 4: Adjusting the prior on the theoretical allele number

To offer some control over the prior distribution on the number of theoretical alleles without risking much accuracy we allow a range of values for *τ *[0.05,0.3]. Table 5 indicates how the default (0.2), minimum (0.05) and maximum (0.3) *τ *values affect results for the one-to-one alignment format using the real data project markers. Results are presented for four sets of real data project markers with the following attributes under the default *τ *a) top alignment *A*1 having *n *= *n*_*min *_+ 1 theoretical alleles, b) the second best alignment *A*2 having a competitive posterior probability to *A*1 and *n *= *n*_*min *_+ 1 theoretical alleles, c) incorrect alignments, and d) a marker with a correct alignment under the default *τ *that sometimes had an inaccurate alignment for the minimum *τ*.

**Table 5:** Results for ten markers aligned with the one-to-one format for three different priors on the number of theoretical alleles.

		# Bins		Default		Min.		Max.	
Group	Locus	Ds1	Ds2	Pr(A1)	Dist.	Pr(A1)	Dist.	Pr(A1)	Dist.
a.)	D4S1531	8	8	0.962	0	0.987	0	0.975	0
	D4S1578	8	8	0.980	0	0.975	0	0.983	0
	D4S1591	6	6	0.997	0	0.989	0	0.993	0
b.)	D4S1534	10	11	0.601	0	0.849	0	0.491	0
	D4S2909	4	5	0.649	0	0.953	0	0.543	0
	D4S2966	6	7	0.665	0	0.884	0	0.579	0
c.)	D4S1637	7	8	0.353	3	0.431	0	0.395	3
	D4S2380	11	16	0.149	7	0.216	11	0.112	11
	D4S3042	13	16	0.304	10	0.326	12	0.220	8
d.)	D4S1517	9	11	0.593	0	0.926	12	0.557	0

Group a.) consists of markers where the top alignment contained *n*_*min *_+ 1 theoretical alleles, the markers in b.) had a 2nd best alignment that had *n*_*min *_+ 1 theoretical alleles, set c.) consists of markers with incorrect alignments, and d.) was an additional marker found to vary in alignment accuracy for different priors.

The results in Table 5 show that the group a) markers with a top alignment containing *n*_*min *_+ 1 alleles, were virtually unaffected by different *τ *values. The markers in group b) benefited from smaller *τ *values because their competing alignments *A*2 consisted of *n*_*min *_+ 1 alleles. This trend was also true for many other real data markers (not shown). The incorrectly aligned markers had slightly varying alignment accuracy for different *τ*'s, but this could be attributed to chance as the low posterior probability alignments have variable top alignment accuracies for different start seeds.

### Feature 5: Using population allele frequencies to align data sets

Since there were no population allele frequencies available to test this feature, we constructed the following test scenario based on six real data project markers D4S1560, D4S2966, D4S406, D4S3042, D4S1637 and D4S2380 (where the first three were aligned correctly and the latter were incorrectly aligned with the one-to-one format). We selected the larger of the two real data project data sets to represent the population allele frequencies for each marker. When feature 5 is selected, MicroMerge v2 uses the allele frequencies provided in the locus file rather than estimating bin frequencies from the pedigree file. The locus file frequencies were used to generate a simulated pedigree file, and the simulated pedigree file was used in place of the original small data set pedigree file in the merging algorithm. Once the alignments were obtained, the two original data files were combined to produce a merged pedigree file.

We used the larger data set's bin frequencies as population frequencies for the smaller data set. The result was a high posterior probability with perfect alignment in each case (not shown). This was expected because the smaller data set's locus file and simulated pedigree file consisted of the same set of unique bins with very similar frequencies to the larger data set. While this example is admittedly simplistic, it illustrates the potential of this feature. If a set of population allele frequencies for a small data set is similar to the allele frequencies of a larger data set, we can expect that MicroMerge v2 will find an accurate, high posterior probability alignment.

### Feature 6: Aligning multiple data sets

**Figure 6:**
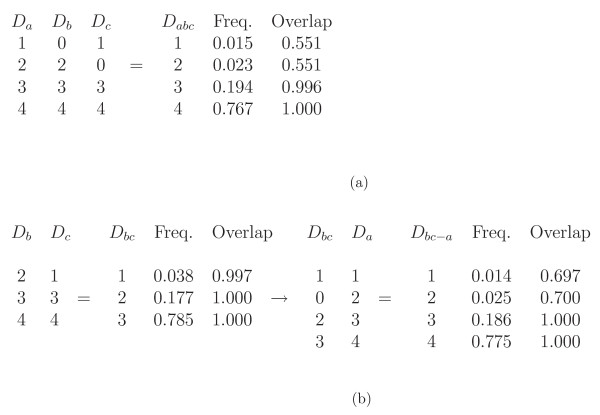
**The multiple data set alignment feature was tested by comparing a) the simultaneous alignment of the three simulated data sets in Table 3, to b) the result from merging data set *b *(*D*_*b*_) and *c *(*D*_*c*_) and then merging this result with the largest data set a (*D*_*a*_).** The theoretical allele frequencies "Freq." and overlap probabilities "Overlap" are provided for each alignment, where bin *i *from lab 1 and a bin *j *from lab 2 overlap if they both align with one or more of the same theoretical alleles. Their overlap probability *o*_*ij *_can be estimated by the fraction of the sampled alignments where overlap occurs. This figure illustrates the importance of merging all data sets simultaneously rather than conducting a series of pair-wise merges. (a) Simultaneous alignment of all three data sets gave the correct alignment with posterior probability 0.55. This posterior probability is lower than the posterior probability for the alignment presented in part b) shown below because the posterior probability of the alignment of *D*_*b *_with *D*_*a *_was low (0.509). (b) The alignment of data set *b *with data set *c *was incorrect, but MicroMerge finds a high posterior probability for their alignment (0.997) because the bin frequencies match well. Since this alignment *D*_*bc *_was not accurate, the alignment of *D*_*bc *_with *D*_*a *_was also inaccurate (posterior probability 0.697).

Results for aligning three simulated data sets are shown in Figure 6. Data set *a *(*D*_*a*_) has two rare bins, 1 and 2, and the two smaller data sets *D*_*b *_and *D*_*c*_each observed one of these bins. When the three data sets were aligned in one MicroMerge v2 run (Figure 6a), the correct alignment was obtained with an acceptable posterior probability. Figure 6b shows that if the data sets were merged using two MicroMerge runs, ie first merging *D*_*b *_and *D*_*c *_and then merging this result *D*_*bc *_with *D*_*a*_, the resulting alignment would be incorrect. This is because merging *D*_*b *_with *D*_*c *_ignores the information available in *D*_*a*_. These results show that aligning three or more data sets in one MicroMerge v2 run has the potential to achieve a more accurate alignment than conducting a series of pairwise merges.

### Feature 7: Likelihood ratio test for assessing alignment quality

The likelihood ratio (LR) test result was useful for the lumped alignment format to evaluate genotype data quality and alert the user to control file problems. This statistic tested the null hypothesis that the unmerged model fit the data equally as well as the merged model. There are three columns in the output summary file related to this statistic. For each marker, the actual LR test statistic is reported, along with the chi-square test statistic for the 0.01 significance level, and then a column indicating rejection (1) or failure to reject (0) the null hypothesis. While posterior probability was generally the best quality measure for individual markers in the real data project, the LR statistic correctly flagged marker D4S1517 as incorrect.

The LR statistic gives a useful indication that the alignment process has run smoothly. In a successful set of MicroMerge v2 runs using default parameters for our 48 real data project markers, 92% had alignments that rejected the null hypothesis. When we ran this same set of markers with 30,000 iterations (where the default run length was 1,000,000), drawing 500 samples (default = 1,000) and the maximum *τ*, only 85% of alignments rejected the null hypothesis. Under these same sampling conditions, but now setting *τ *to the minimum value, the percentage rejecting the null dropped to 75%. As the new MicroMerge v2 features enable more control over the alignment algorithm, it is useful to have additional assurance from the likelihood ratio test that the specified alignment options are appropriate.

### Association analysis of familial dyslipidemia study samples

Table 6 shows the results for Mendel's gamete competition analysis for the 10 chromosome 11 microsatellite markers from the familial dyslipidemia study samples: *Dutch*, *Finn*1 and *Finn*2, where the data sets were analyzed separately and combined. MicroMerge v2 was used to merge all pair-wise combinations and then all three data sets together in both the a.) lumped and b.) one-to-one alignment formats. Table 6 shows that for the individual analyses of the Finnish data sets markers D11S2002 (*Finn*2 P-value = 0.038) and D11S1998 (*Finn*1 P-value = 0.016) were moderately significant. The one-to-one format is generally preferable for data sets with the same ethnicity and reliable genotyping because its assumption that each unobserved allele is missing (as opposed to being lumped into an adjacent allele) is justified in this case. When the two Finnish data sets were merged using the one-to-one alignment format, the D11S2002 association improved from a P-value of 0.038 in *Finn*2 to a merged *Finn*1 - *Finn*2 P-value of 0.011 while the D11S1998 association diminished from a P-value of 0.016 in *Finn*1 to a merged *Finn*1 - *Finn*2 P-value of 0.273. It appears that the significance of the D11S2002 association is largely attributed to its significance in the *Finn*2 data set, as the *Finn*1 P-value for this marker was 0.987. This result illustrates the power gained to detect an association when data sets are merged prior to analysis. Because MicroMerge aligns data sets based on allele frequencies, we have more confidence in MicroMerge's ability to align the two Finnish data sets than to align the Finnish data sets with the Dutch data set where the allele frequencies may be more variable. In the lumped format of the two merged Finnish data sets the D11S2002 association was lower (P-value = 0.109) in comparison to the single data set analysis of *Finn*2 (P-value = 0.038), but it still had the highest association among the ten markers. As a result, D11S2002 appeared to be the best candidate for familial dyslipidemia association among these markers.

**Table 6:** Mendel's gamete competition analysis P-values for three data sets (*Dutch*, *Finn*1 and *Finn*2) analyzed separately and combined using the a) one-to-one and b) lumped alignment formats.

a)	Gamete Competition (GC) P-value			1:1 Format Results: GC P-value and Pr(*A*_1_)			
Locus	*Dutch *(D)	*Finn*1 (F1)	*Finn*2 (F2)	i) D-F1-F2	ii) F1-F2	iii) D-F1	iv) D-F2

D11S1984	0.118	0.191	0.717	0.314 (0.59)	0.689 (0.89)	0.188 (0.65)	0.487 (0.99)
D11S2362	0.318	0.442	0.169	0.127 (0.77)	0.181 (0.57)	0.914 (0.55)	0.057 (0.89)
D11S1999	0.665	0.760	0.965	0.790 (0.90)	0.925 (0.82)	0.636 (0.61)	0.900 (0.64)
D11S1981	0.235	0.477	0.675	0.781 (0.40)	0.801 (0.54)	0.548 (0.44)	0.796 (0.61)
D11S1392	0.614	0.599	0.102	0.068 (1.00)	0.140 (1.00)	0.827 (1.00)	0.035 (1.00)
**D11S2002**	**0.424**	**0.987**	**0.038**	**0.022 (0.27)**	**0.011 (0.97)**	**0.468 (1.00)**	**0.032 (0.97)**
D11S2000	0.843	0.373	0.132	0.694 (0.22)	0.409 (0.49)	0.889 (0.65)	0.414 (0.52)
D11S1998	0.090	0.016	0.859	0.044 (0.65)	0.273 (0.94)	0.010 (0.94)	0.148 (0.58)
D11S4464	0.467	0.132	0.302	0.102 (0.30)	0.142 (0.86)	0.080 (0.27)	0.289 (0.88)
D11S912	0.567	0.099	0.559	0.366 (0.69)	0.286 (0.42)	0.572 (0.96)	0.557 (0.42)

b)	Gamete Competition (GC) P-value			Lumped Format Results: GC P-value and Pr(*A*_1_)			

Locus	*Dutch *(D)	*Finn*1 (F1)	*Finn*2 (F2)	i) D-F1-F2	ii) F1-F2	iii) D-F1	iv) D-F2

D11S1984	0.118	0.191	0.717	0.388 (0.97)	0.508 (0.73)	0.255 (0.62)	0.511 (0.96)
D11S2362	0.318	0.442	0.169	0.137 (0.32)	0.248 (0.32)	0.870 (0.29)	0.050 (0.43)
D11S1999	0.665	0.760	0.965	0.620 (0.64)	0.925 (0.87)	0.542 (0.32)	0.755 (0.71)
D11S1981	0.235	0.477	0.675	0.790 (0.17)	0.438 (0.48)	0.565 (0.27)	0.848 (0.65)
D11S1392	0.614	0.599	0.102	0.068 (0.97)	0.140 (0.93)	0.827 (0.98)	0.035 (0.97)
**D11S2002**	**0.424**	**0.987**	**0.038**	**0.030 (0.34)**	**0.109 (0.73)**	**0.264 (0.52)**	**0.020 (0.67)**
D11S2000	0.843	0.373	0.132	0.459 (0.07)	0.496 (0.38)	0.861 (0.55)	0.478 (0.16)
D11S1998	0.090	0.016	0.859	0.044 (0.52)	0.273 (0.93)	0.010 (0.87)	0.173 (0.50)
D11S4464	0.467	0.132	0.302	0.101 (0.40)	0.230 (0.45)	0.082 (0.25)	0.246 (0.84)
D11S912	0.567	0.099	0.559	0.498 (0.57)	0.415 (0.42)	0.572 (0.67)	0.606 (0.19)

The posterior probability of the top alignment for each marker is included in parentheses. Marker D11S2002 is emphasized because its association significance improved in the one-to-one merge of the two Finnish data sets. This marker was recently discovered to have the strongest linkage among these markers to familial dyslipidemia in a larger fine-mapping analysis.

Interestingly, for the pairwise alignments between the Dutch and Finnish data sets (*Dutch *- *Finn*1 and *Dutch *- *Finn*2) the D11S2002 association was stronger for the lumped alignment format (P-values = 0.264 and 0.020) than the one-to-one format (P-values = 0.468 and 0.032). One explanation is that the differences in allele frequencies between the Dutch and Finnish ethnic groups is more consistent with the lumped format theory. That is, rather than unobserved alleles being missing, they are explained by genotyping differences. These results indicate that the lumped format may be desirable for combining data sets of differing ethnicity.

In practice, it is best to use MicroMerge to align data sets containing samples from the same ethnic group. However, MicroMerge v2's **Feature 5: Using population allele frequencies to align data sets **can offer an effective solution if the appropriate population allele frequencies are available. Furthermore, the posterior probability can alert the user to alignment errors.

## Conclusion

Tools for increasing power in association analysis are becoming increasingly important for complex disease research. MicroMerge software enables a higher-powered analysis of multiallelic marker data projects containing genotype data generated from different laboratories, platforms or protocols. Here we describe extensions to MicroMerge that increase its flexibility and accuracy for merging genotype data sets. We added seven new features to MicroMerge v2. **Feature 1: One-to-one alignment format **creates output files analyzable by most genetic analysis software. While in the real data project the one-to-one format had fewer alignment errors than the lumped alignment format, this is likely because it was a more appropriate format for this test data. Both data sets had fewer than 300 samples, and one data set was about 60% smaller than the other data set. As a result, differences in observed bins between these data sets was likely due to missing alleles in the smaller data set. We recommend using the lumped format if the user intends to analyze the resulting data sets with Mendel software and the data sets meet one of the following four criteria: 1.) only a small percentage (<5–10%) of unique data set bins have rare frequencies, where we define a rare bin as having five or fewer instances in any one data set; 2.) most of the markers have different numbers (differing by two or more) of unique bins between any pair of data sets; 3.) genotyping was done on platforms with different resolving power and 4.) other situations where bin frequencies do not match well. Otherwise we recommend using the MicroMerge v2 default one-to-one alignment format as it performed better on our test data sets and produces more flexible output files.

**Feature 3: Re-merging markers with low posterior probabilities **identifies low confidence regions of an alignment, zeroes rare bins within these regions, and re-merges the adjusted pedigree files. This feature recovers data from markers that might otherwise be discarded due to low confidence alignments because low posterior probability alignments are typically due to a few rare bins.

For markers where the expected alignment should contain *n *= *n*_*min *_alleles, the user can quantify this preference using **Feature 4: Adjusting the prior on the theoretical allele number**, which allows adjustment of the *n *prior parameter *τ*. This feature may be useful when one data set is several times larger than the other data set(s), as the larger data set likely observes all alleles that were present in the smaller data set. Also, in a mixed data set containing both multiallelic and SNP markers this option could obviously be useful for the SNP markers since two alleles are expected. Adjusting the prior to strengthen emphasis on fewer theoretical alleles would improve alignment posterior probability in the case where a particular SNP marker's minor allele frequencies differed by several-fold between data sets.

**Feature 5: Using population allele frequencies to align data sets **should be useful for situations where bin frequencies estimated from the data are unreliable. This feature could be used for small data sets, family data, or to align data sets typed on different ethnic groups. For example, consider a genotyping laboratory *X *with data available on both ethnic groups *A *and *B *for the same marker set. If a research project had genotyped samples with ethnicity *A *at laboratory *X *and wished to combine these samples with a set of ethnicity *B *genotyped at another laboratory *Y*, the *X *laboratory might be able to provide the ethnicity *B *bin frequencies corresponding to ethnicity *A*'s bins. Using the *B *frequencies to represent the *A *pedigree file data would likely improve the alignment of these data sets.

Association analysis of two merged Finnish data sets gave stronger evidence for association than tests conducted on each data set individually. Marker D11S2002 had a moderately significant P-value of 0.038 in *Finn*1 and a P-value of 0.011 in the combined Finnish data sets under the one-to-one alignment option. Indeed, linkage near this locus was found recently in a fine-mapping study (unpublished results). The association test P-value for the lumped alignment merge of the two data sets was 0.109, which was the most significant P-value among the ten markers. Since the one-to-one alignment format uses more information and we were confident in the allele frequency agreement between the two Finnish data sets, it is reasonable that the one-to-one format outperforms the lumped alignment format in this case.

Table 6 indicates that merge iii) combining data sets *Dutch *and *Finn*1 and merge iv) combining *Dutch *and *Finn*2 data sets had more significant P-values for marker D11S2002 associations in the lumped format (P-values = 0.264 and 0.020) than the one-to-one format (P-values = 0.468 and 0.032). Since it is likely that allele frequencies will differ more between the Finnish and Dutch data than the two Finnish data sets, in this case a more conservative alignment format may be appropriate. However, it is better to avoid merging data sets that contain samples with different ethnic origins unless population allele frequencies are available for each group (as described in the **Feature 5: Using population allele frequencies to align data sets **subsections). Even then one must carefully check and correct for any population stratification in the resulting merged data set prior to conducting an association analysis.

MicroMerge was designed to merge data sets of multiallelic markers, which are more difficult to align than SNP data sets due to the greater number of alleles. The MicroMerge alignment algorithm preserves the allele fragment size order indicated in each locus file, which is not directly applicable to SNP data. However, MicroMerge can be used to merge SNP data sets if the major and minor alleles are ordered in the same way in both sets. The correspondence between the alleles of two data sets for a SNP marker is usually known, since the markers are designed to detect specific nucleotides. However, there are several situations in which the application of MicroMerge to SNP data would be of value. **I. Merging recoded alleles: **The most common SNP markers are biallelic, with the two possible nucleotide substitutions known. The SNP allele can be reported as the nucleotide base observed. However, SNP data may be recoded in a variety of ways. SNPs in taqman data files are often coded by the SNP probe dye label, e.g., FAM or VIC, which may need to be aligned to nucleotide codes from another data set. In addition, if primer probes for a SNP are redesigned during the course of a study, it is possible that the dye label may be assigned to the opposite allele in the new probe. Another common coding system is using 0, 1, and 2 to designate the major allele homozygote, the minor allele homozygote, and the heterozygote, not always in the same combination. A third difference in SNP allele calling in different labs could be a result of differences in primer design. If the SNP primer in one lab is designed to anneal to the opposite DNA strand, the resulting SNP nucleotide will be the base complementary to the nucleotide on the strand in the original design. In any of these cases, where the alleles are called correctly but named differently in the two data sets, it is a fairly straightforward task to use the allele frequencies to identify the corresponding major and minor alleles between the two datasets. However MicroMerge can simplify and streamline this task, particularly for large SNP data sets which can number in the hundreds of thousands to millions of SNP markers. **II. Error detection/quality control: **Because MicroMerge gives an alignment posterior probability for every merged allele, using MicroMerge to merge SNP data sets even when it is believed that the correspondence between the alleles is known can identify errors or poor quality data. Because MicroMerge uses allele frequencies to merge alleles, and assigns a confidence for each merge, any problems in the data that result in poorly matched frequencies between the data sets would give low posterior probabilities. These problems could include the following: 1) genotype miscalls, for example those due to errors in clustering by allele calling software allele, 2) different naming schemes between the datasets, for example due to primers designed to opposite strands or probes labeled with different dyes, 4) population stratification between or within data sets. MicroMerge can be applied as a quality control step within a lab, to check between data sets generated at different times or on different platforms. While testing Hardy-Weinberg equilibrium would catch many of these frequency errors, errors might escape detection in the case where a small data set is merged with a large data set. In this situation, the large data set's allele frequencies would likely mask any frequency errors from detection by Hardy-Weinberg analysis performed on the merged data set. MicroMerge would flag frequency differences between the data sets as alignments with poor posterior probabilities. Error detection at each marker is particularly critical for association analysis since a true signal will often not extend beyond a single marker. We plan to illustrate the value of MicroMerge for merging large-scale SNP data sets and flagging potential errors in a future publication.

We have added seven useful new features to MicroMerge v2, and tested them on real and simulated data. In addition, we have used MicroMerge v2 to merge three previously published familial dyslipidemia study samples that were analyzed separately for association. MicroMerge confirmed a genetic association with familial dyslipidemia in the FD study sample. These results show that MicroMerge can effectively increase statistical power in genetic association analysis. MicroMerge v2 software can be run with a simple command line interface. It is available with supporting documentation at  [see Additional file 1].

## Availability and requirements

**Project name: **MicroMerge

**Project home page: **

**Operating system(s): **Linux (any version) on Intel and Windows (Win2K+) on Intel

**Programming language: **Fortran 90

**Other requirements: **none

**License: **none

**Any restrictions to use by non-academics: **none

## Authors' contributions

APP, EMS and JCP conceived of and developed most of the MicroMerge v2 features. DEW and KÅ developed the one-to-one alignment method and debugged the software. PP and CP provided the familial dyslipidemia data. The manuscript was written by APP and all authors revised and approved the manuscript.

## Supplementary Material

Additional file 1 MicroMerge v2 software for Windows. This file contains a copy of MicroMerge v2 software for Windows (Win2K+) on Intel. Versions of MicroMerge v2 developed for Linux and Macintosh systems, as well as current releases of MicroMerge can be found here: . Click here for file
