# Supplementary material for: Merging microsatellite data: enhanced methodology and software to combine genotype data for linkage and association analysis
Source: BMC Bioinformatics. 2008 Jul 21;9:317. doi: 10.1186/1471-2105-9-317 (PMC2515855; doi:10.1186/1471-2105-9-317)
Supplement: Additional file 1 — MicroMerge v2 software for Windows. This file contains a copy of MicroMerge v2 software for Windows (Win2K+) on Intel. Versions of MicroMerge v2 developed for Linux and Macintosh systems, as well as current releases of MicroMerge can be found here: . [file 1471-2105-9-317-S1.zip › MicroMerge_v20_Windows/MicroMerge_Input-File_Specifications_2007.pdf]

MicroMerge Input File Specifications

Angela Presson
apresson@ucla.edu

September 21, 2007

Overview

- MicroMerge requires a minimum of five input files:
  - Mendel locus file for each data set to be merged (2 or more files).
  - Mendel pedigree file for each data set to be merged (2 or more files).
  - MicroMerge control file (1 file).
- In addition to the required files there are two optional files:
  - MicroMerge inclusion status file (1 file).
  - MicroMerge samples in common file (1 file).
- The locus and pedigree files must be in comma-separated Mendel format, with one notable exception: MicroMerge allele names are limited to 3 characters. Some format information is given below in the 'Locus File' and 'Pedigree File' sections, but more details on constructing these files can be found in the mendel manual (http://www.genetics.ucla.edu/software/).
- The MicroMerge control file differs from the Mendel control file, and the format is provided below in the 'Control File' section.
- The optional comma-separated inclusion status file indicates which loci will be merged and enables locus-specific genotype error rates.
- The locus, pedigree, and inclusion status files must contain the same loci in the same order (ie they must be coordinated).
- A samples in common file should be included if there are samples that have been genotyped in more than one data set.
  - It must contain pedigree and individual ID's for each sample, and there must be two columns for each data set listed in the Control file.
  - The data set column order must be coordinated with the data set row order in the control file.
- MicroMerge requires locus files to have a maximum line length of 256 characters and limits pedigree files to 65,000 characters per line. The inclusion status file has a maximum length of 256 characters and the control and samples in common files have a limit of 512 characters.

Locus File

- Identical to a comma-separated Mendel version 7 locus file, except that allele names are limited to three characters.
- The alleles for each locus are listed in either ascending or descending size order.
- The allele ordering system (ascending or descending) must be the same in each locus file.
- Marker names are limited to 16 characters.

Pedigree File

- Identical to a comma-separated Mendel pedigree file, except that allele names are limited to three characters and alleles are separated by a dash -.
- All data must be genotypes except at a trait locus.
- Comma-separated Mendel format:
  - Blanks for missing values.
  - The first six columns in pedigree file include the following individual information: pedigree ID, individual ID, parent 1 ID, parent 2 ID, sex, and twin status, where each field is limited to 10 characters except the pedigree ID, which is limited to 7 characters.
  - The genotype data starts in column 7 and each genotype has a maximum length of 7 characters.

Control File

- This file, which should be named "control.txt", is similar to the Mendel control file (but the key words are ofcourse different) in that each key word is followed by an equals sign = , and a value (except for the PEDIGREE_AND_LOCUS_FILES parameter, which has a unique format).
- The minimum requirement for this file is the keyword PEDIGREE_AND_LOCUS_FILES, specifying at least two pairs of locus and pedigree files to be merged. Exclusion of all other keywords results in default values.
- See Table 1 for a list of all keywords, followed by their descriptions.

Inclusion Status File (Optional)

The inclusion status file is optional and unique to the MicroMerge program. It is specified in the control.txt file with the keyword INCLUSION_STATUS_FILE. If there is no inclusion file, then each locus is merged using the default genotype error rate (which can be changed in the control file). The format for this file is one marker per line followed by a comma and an inclusion status. The following inclusion status values are understood:

- Blank = merge this locus with the default error rate.
- A number in the range (0,1) = merge this locus with the indicated error rate.
- 0 = merge this locus with no genotyping error.
- -1 = do not merge this locus but print its (input file) data in the merged output files.
- -2 = do not merge this locus and exclude it from the output files.

Samples in Common File (Optional)

- This file is optional but should be included if there are samples that have been typed in all of the data sets to be merged.
- Each row contains the pedigree and individual ID's for one sample.
- The number of columns corresponds to the number of data sets times two.
- The column order should be coordinated with the order in which the pedigree and locus files are listed in the control file.

  For example, a pair of data sets with four samples typed in common could have the following samples in common file data:

  PED11, IND323, PED42, IND452

  PED11, IND325, PED42, IND453

  PED31, IND443, PED92, IND714

  PED21, IND404, PED32, IND213

Keywords

1. PEDIGREE_AND_LOCUS_FILES: This is the only keyword required in the control.txt file. Starting on the line following this keyword, list the names of the pedigree and locus files for each data set (maximum length of 128 characters). Each line should contain a pedigree and locus file for one data set, where the file names are separated by a comma. At least two data sets must be included. For example:

   PEDIGREE_AND_LOCUS_FILES =

   pedigree1.txt,locus1.txt

   pedigree2.txt,locus2.txt

   pedigree3.txt,locus3.txt

2. SAMPLES_IN_COMMON_FILE: You should include this file when samples have been genotyped in each of the data sets that will be merged. See the section “Samples in Common File (Optional)” for details on creating this file.

3. INCLUSION_STATUS_FILE: Including this file enables control of which markers will be merged, printed, or excluded from the output files. It also allows for marker-specific genotyping error rates. If this keyword is omitted, MicroMerge assumes that all markers should be merged with a genotyping error rate 0.02 or an alternate rate specified by keyword GENOTYPING_ERROR. See the section “Inclusion Status File (Optional)” for details on creating this file.

4. ONE_TO_ONE_ALIGNMENT: MicroMerge can produce merged data files analyzable by Mendel (by setting this keyword to 0, or omitting it), or it can create files that are compatible with most genetic analysis packages based on a less conservative alignment approach (by setting this keyword to 1).

5. START_SEED: Setting this keyword to 0 tells MicroMerge to pick a random seed, omitting it instructs MicroMerge to use the default seed 12181. The user can also specify a seed (five character maximum).

6. ITERATIONS: This keyword sets the number of MCMC iterations for each marker. The default run length is 1000000 (10^6); but in cases where markers contain many alleles (> 20), a longer run might improve convergence and alignment quality. Note however that runs longer than 5000000 (5 x 10^6) iterations may exceed 24 hours!

7. RUN_MAXIMUM: This keyword may be helpful if the user wishes to avoid the automatic (and recommended) iteration increase for smaller ALLELE_SET_SIZE values. It accepts integer values (of 10 characters or less) $\geq$ to the ITERATIONS keyword value. The default is 15000000 (1.5 x 10^7).

8. BURN_IN: Allows control over the number of iterations that are discarded before sampling, which should be approximately 0.1-1% of the number of iterations. The default value is 1000 (10^3).

9. SUPPRESS_ERRORS: A value of 1 suppresses warning messages. The default is 0.

10. GENOTYPE_ERROR: This keyword specifies a genotyping error rate for all markers, incorporating mistyping in the likelihood calculation. It accepts real numbers in the range [0.0-1.0] with a 7 character maximum and a default value of 0.02. Control of marker-specific genotyping error rates is enabled via the INCLUSION_STATUS_FILE.

11. ALLELE_SET_SIZE: Controls the emphasis on the number of theoretical alleles proposed. This keyword accepts real numbers in the range [0,1] and has a default value of 0.5. Values less than the default put stronger emphasis on theoretical allele values near the minimum, while values greater than the default, though still emphasizing theoretical allele values near the minimum, are less demanding. Because values less than 0.5 increase convergence time, MicroMerge automatically increases the number of MCMC iterations. (Note that the user can override this response by setting the RUN_MAXIMUM equal to the desired number of iterations.)

This keyword is useful for the situation where the user has confidence that one data set includes all other alleles observed in the second data set. For example if one data set contains several times more samples than the other data set, and the latter data set is also fairly small resulting in notable discrepancies in allele frequencies. A run with the default value may result in an alignment with an extra allele(s) because the allele frequency discrepancies lead MicroMerge to believe that there is either a missing allele(s) from both data sets or genotyping differences. By selecting an ALLELE_SET_SIZE value smaller than 0.5, for example 0.3, MicroMerge could find the more likely alignment with the number of theoretical alleles equal to the minimum.

12. MIN_POSTERIOR_PROBABILITY: This keyword, along with keywords PAIRWISE_-PROBABILITY_THRESHOLD and RARE_ALLELE_THRESHOLD, controls the tendency to re-merge markers that have weak alignments. MIN_POSTERIOR_PROBABILITY is a real number within the range [0.0,1.0] with a maximum of 7 characters, and a default value of 0.425 for lumped alignments and 0.575 for one-to-one alignments. It specifies the minimum posterior probability that must be attained for a marker alignment to 'pass' and not require re-merging. If the posterior probability for the alignment of a particular marker falls below this threshold, MicroMerge then checks for weak parts of the alignment, by examining the pairwise probabilities and theoretical allele frequencies. If there are low pairwise probabilities (where the threshold is governed by keyword PAIRWISE_PROBABILITY_THRESHOLD) and additionally, the corresponding theoretical allele frequencies for these low probability pairs are rare (governed by RARE_ALLELE_THRESHOLD), MicroMerge will zero the data set bins corresponding to the rare theoretical alleles and then re-merge the marker data. If the user does not wish for MicroMerge to re-merge markers, this value can be set to 1.0.

13. PAIRWISE_PROBABILITY_THRESHOLD: Accepts a real number within the range [0.0,1.0] with a maximum of 7 characters and a default value 0.85. See keyword MIN_POSTERIOR_-PROBABILITY for an explanation of this keyword's involvement in MicroMerge's re-merging feature.

14. RARE_ALLELE_THRESHOLD: Accepts a real number within the range [0.0,1.0] with a maximum of 7 characters and a default value 0.015. See keyword MIN_POSTERIOR_-PROBABILITY for an explanation of this keyword's involvement in MicroMerge's re-merging feature.

15. NUMBER_SAMPLED: Controls the number of alignments sampled from the posterior distribution. The default is 1000 corresponding to the default number of iterations. While more samples may improve accuracy, it is important that there are enough intermediate iterations between samples that they are independent. As a result, it is recommended that this value only be increased in conjunction with increasing the number of iterations.

16. POPULATION_FREQUENCIES: This keyword accepts a vector with integer values and length equal to the number of data sets. Its default value is 0,0,...,0 indicating that MicroMerge should estimate allele frequencies from the pedigree files. A value of 1 indicates that MicroMerge should simulate pedigree data based on the allele frequencies in the corresponding locus file (with a sample size chosen by MicroMerge). For example, POPULATION_FREQUENCIES = 0,1,0 would indicate that the second data set should be merged using the allele frequencies in its locus file, while the first and third should be merged using

the allele frequency estimates from their pedigree files. The user can control the sample size for the simulated pedigree file by specifying an integer value other than 1. For example, POPULATION_FREQUENCIES = 0,650,0 would indicate that the simulated pedigree file for the second data set should contain 650 samples.

17. SIMULATE_DATA_INSTRUCTIONS: Use with keyword POPULATION_FREQUENCIES to choose from the following three pedigree file simulation options: 1.) setting this keyword to 0 indicates that MicroMerge should cease execution after generating a simulated pedigree file, 2.) 1 indicates that data should be simulated by sampling alleles from population frequencies given in the locus file and then the data files should be merged using this simulated data, and 3.) 2 tells MicroMerge to simulate data by computing the exact number of genotypes under HWE from the population frequencies given in the locus file and then to merge the data sets using this simulated data. Simulated data files are given the following name(s): "dataset_generated_ped_MM-DD-YY.txt".

18. NUMBER_OF_TRANSLATIONS: This keyword is only applicable when the output files will be in the one-to-one alignment format (ONE_TO_ONE_ALIGNMENT = 1). It controls the number of one-to-one translations corresponding to each sampled lumped alignment, accepting integer values with a default of 1. See the publication corresponding to MicroMerge version 2 for details.

Table 1: MicroMerge v. 2.0 keyword list.

	Keyword	Default	Type	Definition
1.	PEDIGREE_AND_LOCUS_FILES		128 Char	Pedigree and locus file names
2.	SAMPLES_IN_COMMON_FILE		128 Char	Name of samples in common file
3.	INCLUSION_STATUS_FILE		128 Char	Name of inclusion status file
4.	ONE_TO_ONE_ALIGNMENT	0	0/1	Mendel compatible or general files
5.	START_SEED	12181	Int (5 Char)	MCMC start seed
6.	ITERATIONS	1000000	Int (10 Char)	Number of iterations for MCMC loop
7.	RUN_MAXIMUM	15000000	Int (10 Char)	Maximum number of iterations
8.	BURN_IN	1000	Int (8 Char)	Runs discarded before sampling
9.	SUPPRESS_ERRORS	0	0/1	Default suppresses warning messages
10.	GENOTYPE_ERROR	0.02	Real (7 Char)	Genotyping error rate
11.	ALLELE_SET_SIZE	0.5	Real in [0,1]	Option for merging small data set(s)
12.	MIN_POSTERIOR_PROBABILITY	0.425/0.575	Real in [0,1]	Threshold for re-merge
13.	PAIRWISE_PROBABILITY_THRESHOLD	0.85	Real in [0,1]	Threshold for re-merge
14.	RARE_ALLELE_THRESHOLD	0.015	Real in [0,1]	Threshold for re-merge
15.	NUMBER_SAMPLED	1000	Int (8 Char)	Number of MCMC samples to save
16.	POPULATION_FREQUENCIES	0,0,...	Integer	Option for merging small data set(s)
17.	SIMULATE_DATA_INSTRUCTIONS	1	0/1/2	Instructions for handling small data sets
18.	NUMBER_OF_TRANSLATIONS	1	Int	Per alignment translations from lumped format
19.	RE-MERGED_PREFIX	"RE-"	8 Char	Prefix for re-merged file names
20.	MERGED_PEDIGREE_FILE_NAME	"Merged_Pedigree_MM-DD-YY.txt"	128 Characters	Merged pedigree file name
21.	MERGED_LOCUS_FILE_NAME	"Merged_Locus_MM-DD-YY.txt"	128 Characters	Merged pedigree file name
22.	MERGED_SUMMARY_FILE_NAME	"Merged_Summary_MM-DD-YY.txt"	128 Characters	Merged pedigree file name
23.	MERGED_LOG_FILE_NAME	"Merged_Log_MM-DD-YY.txt"	128 Characters	Merged log file name
